# Management of heart failure with preserved ejection fraction in a local public hospital in Hong Kong

**DOI:** 10.1186/s12872-015-0002-8

**Published:** 2015-02-25

**Authors:** Angel W Leung, Cherise Y Chan, Bryan P Yan, Cheuk Man Yu, Yat Yin Lam, Vivian W Lee

**Affiliations:** School of Pharmacy, Faculty of Medicine, The Chinese University of Hong Kong, Shatin, Hong Kong; Department of Medicine and Therapeutics, Faculty of Medicine, The Chinese University of Hong Kong, Shatin, Hong Kong

**Keywords:** Heart failure with preserved ejection fraction, Direct medical cost, Drug use pattern, Quality of life

## Abstract

**Background:**

Heart failure (HF) is one of the most debilitating chronic illnesses. The prevalence is expected to increase due to aging population. The current study aimed to examine the management of heart failure with preserved ejection fraction (HFpEF) including drug use pattern, direct medical cost and humanistic outcome in a local public hospital in Hong Kong.

**Methods:**

The current study adopted the retrospective observational study design. Subjects were recruited from the Heart Failure Registry of the Prince of Wales Hospital in Hong Kong between 2006 and 2008 and completed the Minnesota Living with Heart Failure Questionnaire (MLHFQ) at 3 designated time-points conferred eligibility. Patients with significant valvular disorder were excluded. Each patient’s medical record was reviewed for 12 months after the date of admission. Heart failure related admissions, clinic visits, cardiovascular drugs, laboratory tests and diagnostic tests were documented. Costs and MLHFQ scores in patients with or without hypertension, diabetes and renal impairment were compared.

**Results:**

A total of 73 HFpEF patients were included. It was found that loop diuretics (93.1%, 78.1%) was the most frequently used agent for HFpEF management in both in-patient and out-patient settings. The mean 1-year direct medical cost was USD$ 19969 (1 US $ = 7.8 HK$), with in-patient ward care contributing to the largest proportion (72.2%) of the total cost. Patients with diabetes or renal impairment were associated with a higher cost of HFpEF management. Significant difference was found in the renal impairment group (median cost: USD$ 24604.2 versus USD$ 12706.8 in no impairment group, p = 0.023). The MLHFQ scores of the subjects improved significantly during the study period (p < 0.0005).

**Conclusions:**

The cost of management of HFpEF was enormous and further increased in the presence of comorbidities.

## Background

Heart failure (HF) is one of the most debilitating chronic illnesses in Hong Kong, especially in the elderly. With an overall incidence of 0.7 per 1000 population in 1997 [1], the number of cases of heart failure hospitalizations has been tripled over the past 10 years [2]. In 2009, heart failure was the cause of death of 794 patients in Hong Kong [3]. Heart failure with preserved ejection fraction (HFpEF), a sub-division of heart failure, constitutes 40-50% of heart failure cases [4,5]. When compared to heart failure with reduced ejection fraction (HFrEF), HFpEF has a slightly lower mortality [6] but similar hospital readmission rate and decline in functional status [5,7]. Nevertheless, studies investigating into the cost and drug use pattern of HFpEF management remain limited. In United Kingdom, the estimated direct cost for the management of HF increased from 1.2% of total National Health Service (NHS) expenditure in 1990–01 to 1.83% in 1995, equivalent to GBP£ 716 million [8]. The estimated cost increased to 1.91% of the NHS expenditure in 2000. The cost management almost doubled in a decade, with 69% of the cost attributed to hospitalization [9]. In Sweden, the estimated annual treatment cost for HF including costs of institutional care, outpatient care, surgery and medications, constituted 2% of the Swedish healthcare budget [10]. In the United States, The estimated direct and indirect costs of HF in 2010 were US $ 24.7 billion and $ 9.7 billion respectively [11].

Given that HF has an age-related prevalence [6], it can be anticipated that the disease will pose an increasing financial burden to Hong Kong in view of the aging population. In addition, the advancement in management of acute cardiovascular disease shifts the mortality pattern to HF. The American Heart Association projects a 200% increase in annual medical cost of HF over 2010–2013 [11]. Hence, the current study aims to investigate the HFpEF management in Hong Kong with the focus of the direct medial cost, drug use pattern and humanistic outcome of HFpEF management.

## Methods

A retrospective non-randomized case review design was adopted in this study. All study subjects were recruited from the Heart Failure Registry of the Prince of Wales Hospital (PWH), the major acute hospital in the New Territories East Cluster (NTEC) in Hong Kong. Their medical records were retrieved from the Clinical Management System (CMS) for the evaluation of the direct medical cost, drug use pattern and humanistic outcome of HFpEF management during the first year after entering the registry. The presence of comorbidities, including hypertension, diabetes mellitus and renal impairment (defined as serum creatinine > 200 umol/L on admission), and other baseline parameters were also recorded. The protocol of this study was approved by the Joint CUHK-NTEC Clinical Research Ethics Committee in October 2011. Patients with (1) signs and symptoms typical of HF, (2) with normal or only mildly reduced left ventricular ejection fraction (LVEF > 40%) and (3) relevant structural heart disease and/or diastolic dysfunction, diagnosed of HFpEF according to the diagnostic criteria of Heart Failure and Echocardiography Associations of the European Society of Cardiology were included. They were in the Heart Failure registry of PWH within the period of 1st January, 2006 to 31st December, 2008, and complete the Minnesota Living with Heart Failure Questionnaire (MLHFQ) at baseline, 3 month and 12 month follow-up visits. Patients with valvular diseases of moderate, severe or unknown severity were excluded.

The usage of all cardiovascular medications during the study period was recorded. Medications under consideration included Angiotensin Converting Enzyme Inhibitors (ACEIs), Angiotensin Receptor Blockers (ARBs), beta-blockers, Calcium Channel Blockers (CCBs), diuretics, aldosterone antagonists, digoxin, antiplatelets, anticoagulants, anti-arrhythmics, nitrates, vasodilators, lipid-lowering agents and potassium supplements. The prevalence of use among study subjects was investigated to reflect the drug use pattern for HFpEF management in PWH in Hong Kong. The direct medical costs for the management of HFpEF were evaluated from the perspective of the Hong Kong Hospital Authority. The total sum consisted of spending in HF-related hospitalizations, outpatient clinic visits and Accident and Emergency Department (AED) admissions. All admissions or consultations in which HF was a principle diagnosis or part of the focus of management were included. The costs of all services, medications and tests were estimated with reference to the Hong Kong Government Gazette 2003 [12] and Hong Kong Hospital Authority Drug Formulary 2006 [13]. The direct medical costs were analyzed to reveal any statistically significant differences between subjects with or without hypertension, diabetes mellitus or renal impairment.

The MLHFQ, a 21-item HF-specific questionnaire, was adopted to evaluate the quality of life of the study subjects. All subjects had completed the Chinese version of the questionnaire at baseline, 3-month and 12-month. A 6-point scale from 0–5 was used in the questionnaire, with 0 representing minimal impact and 5 representing most significant impact of HF on their life during the past 4 weeks. The overall score was a sum of the scores of the 21 questions. The physical domain score and emotional domain score were also calculated. Higher scores indicate poorer quality of life [14].

The prevalence of use of each drug was expressed as the percentage of patients using the drug, while the direct medical cost for the management of HFpEF, MLHFQ scores and other baseline values were presented in mean ± standard deviation and median with interquartile range and range (min-max). Wilcoxon Signed Rank Test was performed to test for statistical significance of the differences in MLHFQ scores at 3-month and 12-month with respect to the baseline. For comparison of the direct medical costs and MLHFQ scores between different comorbidity subgroups, Mann–Whitney Test was adopted. Non-parametric tests were preferred in view of the small sample sizes and the presence of outliers in various cohorts. All statistical analyses were performed using Statistical Package for the Social Sciences (SPSS version 16.0, SPSS Inc.). Differences were considered statistically significant when the p-value is <0.05.

### Ethical approval

The study was approved by the Ethics Committee, CUHK-New Territories East Cluster, Hospital Authority, Hong Kong. (CREC Ref. No: CRE-2011.457). The current protocol was in compliance with the Declaration of Helsinki.

## Results

114 patients in the Heart Failure Registry of PWH were screened for study eligibility. 41 subjects were excluded as they did not meet the inclusion criteria of the study or their medical records were incomplete. A total of 73 HFpEF patients were recruited into this study. Their demographic characteristics and baseline parameters of the subjects were summarized in Table 1. The majority of the subjects were female (69.8%), with an average age of 76.2 years old on admission and average LVEF of 64.3%. Among the three comorbidities of interest, hypertension (76.7%) was the most common, followed by diabetes (46.6%) and renal impairment (16.4%). 22 and 10 patients were comorbid with 2 and 3 of the diseases respectively. The mean length of hospital stay was 23.4 days, with an average of 2.2 admissions per person and a readmission rate of 52.1%. When stratified by the presence of comorbidity, the renal impairment group was found to have the longest duration of hospital day (35.0 ± 32.9 days), highest number of admissions (mean: 2.5 ± 1.0 admission) and readmission rate (83.3%) followed by diabetes and hypertension patients. The most commonly used class of medications was diuretics, with a usage rate of 94.5% and 80.8% under in-patient and outpatient settings respectively. ACEIs (69.9%, 65.8%), antiplatelets (57.5%, 60.3%), beta-blockers (56.2%, 58.9%) and CCBs dihydropyridine (54.8%, 57.5%) were also used in over half of the study subjects under both settings.

**Table 1:** **Demographic characteristics of study subjects**

**No. of subjects**		**N = 73**	
Gender (n, %)			
	Male	22	30.1%
	Female	51	69.9%
Age on admission (n, %)			
	Mean ± SD	76.2 ± 10.3	
	Aged 30-54	4	5.5%
	Aged 55-64	4	5.5%
	Aged 65-74	16	21.9%
	Aged 75-84	37	50.7%
	85 or above	12	16.4%
Social history (n, %)			
	Smoker	6	8.2%
	Ex-smoker	17	23.3%
	Non-smoker	30	41.1%
	Unknown	20	27.4%
Ejection fraction on admission			
	Mean ± SD	64.3 ± 8.9%	
NYHA class on admission (n, %)			
	Class I – II	10	13.7%
	Class III	35	47.9%
	Class IV	19	26.0%
	Unknown	9	12.3%
Comorbidity (n, %)			
	Hypertension^#^	56	76.7%
	Diabetes mellitus^#^	34	46.6%
	Renal impairment^$^	12	16.4%
	Comorbid with 2 of the above	22	30.1%
	Comorbid with 3 of the above	10	13.7%
	Coronary artery disease	25	34.2%
	Atrial fibrillation	17	23.3%
Blood pressure (n = 72)			
• Systolic BP (mean ± SD)		155.9 ± 35.1 mmHg	
• Diastolic BP (mean ± SD)		78.4 ± 19.0 mmHg	
• ≥140/90 mmHg (n, %)		51 70.8%	
• Comorbid with HTN (n = 55), ≥ 140/90 mmHg (n, %)		39 70.9%	
• Comorbid with DM (n = 33), ≥ 130/80 mmHg (n, %)		28 84.4%	
Blood glucose			
HbA1c (n = 32)			
	Mean ± SD	6.7 ± 1.3%	
	>6.5% (n, %)	14	43.8%
	Comorbid with DM (n = 25), > 6.5% (n, %)	14	56.0%
Fasting blood glucose (n = 32)			
	Mean ± SD	7.2 ± 3.3 mmol/L	
	≥7 mmol/L	10	31.3%
	Comorbid with DM (n = 16), ≥ 7 mmol/L (n, %)	9	56.3%
Lipid panel			
LDL (n = 42, mean ± SD)		2.2 ± 1.0 mmol/L	
HDL (n = 42, mean ± SD)		1.4 ± 0.6 mmol/L	
Triglyceride (n = 43, mean ± SD)		1.4 ± 0.6 mmol/L	
Total cholesterol (n = 45, mean ± SD)		4.2 ± 1.3 mmol/L	
Serum creatinine (n = 73)			
	Mean ± SD	142.0 ± 100.7 umol/L	
	>200 umol/L^$$^ (n, %)	12	16.4%
	Mean ± SD of subjects with sCr > 200 umol/L	330.7 ± 111.2 umol/L	

^#^According to physician’s diagnosis.

^$^Defined as baseline serum creatinine > 200 umol/L.

^$$^Regarded as clinically significant renal dysfunction.

The 1-year direct medical costs incurred by the management of HFpEF in the study subjects. The mean and median total costs for HFpEF management were USD$19969 and USD$15178.8 per patient respectively (1 US $ = 7.8 HK$). In-patient care constituted the largest part of expenditure among all categories and contributed to 91.7% of the total spending in HFpEF management, followed by outpatient care and A&E admission. In-patient ward care was the major cost driver within the in-patient category, comprising of 72.2% of the total direct medical costs. The mean and median 1-year direct medical costs per patient for the management of HFpEF in different comorbidity cohorts. Subjects who were comorbid with renal impairment were associated with the highest spendings in HFpEF management, with a mean and median total cost of USD$ 31645.2 and USD$24604.2 respectively. The cost incurred by subjects with diabetes came second, while subjects with hypertension were associated with the least cost for HFpEF management. In general, the cost of care in the presence of comorbidity was higher than that of the overall study subjects.

In the renal impairment group, the median costs of in-patient (p < 0.0005) and out-patient laboratory tests (p = 0.043), overall in-patient cost (p = 0.027), overall out-patient cost (p = 0.019) and total cost (p = 0.023) were significantly higher than those in the control group. For the diabetes group, the in-paitent and out-patient costs for medications (p = 0.009; p < 0.0005) and laboratory tests (p = 0.001; p < 0.0005), and the overall out-patient costs (p < 0.0005) were significantly higher than the non-diabetes mellitus control. The costs of hypertenison group were higher than its control, but none of the cost items reached statistical significance.

Progressive improvements in the total scores, physical domain and emotional domain were observed throughout the 1-year period. When compared to the baseline median scores, both the 3-month and 12-month total scores and physical domain scores improved significantly (p < 0.0005 for all). The emotional domain score also improved significantly at 12-month follow-up when compared to the baseline (p < 0.0005). However, when comparing the scores between the comorbidity cohorts and controls, no statistically significant differences was detected between subjects with any one of the comorbidities and the control groups, with only the renal impairment group showing a significantly worse MLHFQ total scores at baseline (p = 0.046) (Table 2).

**Table 2:** **Minnesota living with heart failure questionnaire scores at baseline, 3-month follow-up and 12-month follow-up by the presence of comorbidity**

		**Hypertension**		**Diabetes mellitus**		**Renal impairment**	
		**Yes**	**No**	**Yes**	**No**	**Yes**	**No**
		**n = 56**	**n = 17**	**n = 34**	**n = 39**	**n = 12**	**n = 61**
**Total scores**							
Baseline							
	Median (IQR)	31 (17.25)	27 (15)	32 (13.75)	27 (19)	34(12.5)	28(18)
Follow-up after 3 months							
	Median (IQR)	21 (15.5)	30 (25)	21 (14)	24 (18.5)	22(9.5)	23(21)
Follow-up after 12 months							
	Median (IQR)	12.5 (12.25)	15 (12)	12 (14.25)	15 (11)	12.5(10.5)	14(13)
**Physical domain**							
Baseline							
	Median (IQR)	18 (7.25)	18 (8)	18.5 (4.75)	17 (10)	20(5.25)	18(8)
Follow-up after 3 months							
	Median (IQR)	11 (9.5)	12 (13)	9 (8.5)	12 (12)	9.5(5)	11(10)
Follow-up after 12 months							
	Median (IQR)	7 (6.5)	9 (5)	7 (8.25)	8 (6.5)	8(6.5)	7(8)
**Emotional domain**							
Baseline							
	Median (IQR)	5 (5)	5 (8)	6 (5.75)	5 (5.5)	7.5(4.75)	5(6)
Follow-up after 3 months							
	Median (IQR)	5 (4)	8 (10)	5 (4.75)	5 (6)	5(4.25)	5(6)
Follow-up after 12 months							
	Median (IQR)	3 (5)	5 (4)	2 (5)	5 (4)	4(4.5)	4(4)

## Discussion

The demographics of the subjects in this study resembled those in previous controlled trials, population-based and registry-based studies conducted locally or overseas [15-17]. The majority of our patients were female, which constituted up to 70% of the study population. The subjects were also at advanced age in general, with a mean age of 76 on admission. This, together with existing studies, suggested that female sex and advanced age are prevalence factors of HFpEF. For the presence of comorbidities at baseline, 77% of the study subjects had a history of hypertension, followed by 47% for diabetes and 16.4% for renal impairment. The proportions matched well with those reported in the ADHERE database [15]. Similar drug use pattern was observed in previous overseas studies by Yancy et al. and Smith et al. in which the frequent use of diuretics, ACEIs and beta-blockers was documented [5,15]. Due to a lack of large randomized trials, the use of medications in DHF is largely guided by inconclusive trial results or expert opinions [14,18-20]. Control of blood pressure, heart rate and blood volume, and prevention of myocardial ischemia were recommended by ACC/AHA and HFSA guidelines [21,22], which were observed locally.

The 1-year direct medical cost of HFpEF management differed among subjects with different comorbidities. The cost involved was the highest in renally impaired subjects and lowest in those with hypertension. When compared with the corresponding control group, statistically significant differences were shown in a number of cost categories in diabetes and renal impairment groups, but not the hypertension group. Hypertension, diabetes and renal impairment are diseases associated with a poorer prognosis in HFpEF [23-25]. The presence of these comorbidities therefore gave rise to extra HFpEF management costs [26]. MacDonald et al. for instance, showed that in a group of HFpEF patients from the CHARM programme, the rate of HF-related hospitalization was significantly higher in the diabetic group than the non-diabetic group [27]. Krumholz et al. also revealed a significantly longer duration of hospitalization and higher in-patient cost in patients developing deteriorated renal condition than those who did not [28]. An increase in the duration of hospital stay certainly increases the costs of in-patient care. Being the major cost driver in our study, this increased the total direct medical cost in these comorbidity cohorts. Apart from the increased in-patient ward care spending, more laboratory examinations might have to be performed with increased complexity of illness.

Given that in-patient care accounts for a major portion of the cost of HFpEF [29,30], measures to reduce LOS and readmission rate can potentially achieve cost-saving. Literatures reported that implementation of multidisciplinary HF management programme improved outcomes, reduced hospitalization and lowered the cost in HFrEF [31-33]. The intervention generally focused on optimizing drug therapy, enhancing patients’ understanding on disease and drugs, and improving compliance on medications and lifestyle (e.g. salt restriction). The abovementioned local HF management programme was implemented in Pamela Youde Netheresole Eastern Hospital in 2001–2003, comprising a team of physicians, registered nurses, physiotherapists, occupational therapists and dietitians [34]. The programme was found to significantly reduce hospitalization, decrease the total number of bed day and achieve an average saving of USD$1453.8 per patient over a 6-month period.

For the humanistic outcome, it was found that the total, physical and emotional MLHFQ scores were reduced with statistical significance after 1 year, indicating an improvement in the quality of life of subjects during the course of HFpEF management. The MLHFQ developers commented a 5-point improvement in the total score as clinically significant [35]. Hence, the MLHFQ score potentially serves as a tool to assess the benefits and risks of a treatment [36-38]. In our study, the subjects on average showed 6.39 and 14.97 points of MLHFQ score improvements at 3-month and 12-month follow-up respectively. As the Chinese version of MLHFQ was previously validated in a Taiwan study [14], the improvement in scores was believed to reflect the effectiveness of the current interventions in maintaining the quality of life of the study subjects.

There were several limitations in the present study. Firstly, the sample size was small. The 73 subjects recruited may not be representative of the majority of HFpEF patients, although the well-matched demographics and baseline parameters provided some evidence of credibility. Secondly, our sample was drawn from the NTEC. The results may not truly reflect the HFpEF management in patients in Hong Kong due to variations in practices and management approaches across clusters. Thirdly, the retrospective design of our study relied on well-documented medical records. Investigations or treatments not properly documented introduced errors to cost estimation. Fourthly, the cost estimation of healthcare services was based on the Hong Kong Government Gazette 2003. The costs were assumed to be applicable to the study period in 2006–2008 as no update on healthcare charges was available, but the effect of inflation was not taken into account. Last but not least, the cost-of-illness was not limited to the direct medical cost from the perspective of a healthcare institution. Long-term care costs, indirect medical costs and intangible costs were not included in our study. Further studies should be directed to a more holistic approach, preferably with cost-effectiveness evaluation on the current HFpEF treatments. This will provide more evidence-based support for local practice, given that no international consensus has been reached regarding HFpEF management.

## Conclusion

The present study evaluated the management of HFpEF patients. Loop diuretics, ACEIs, beta-blockers, dihydropyridine CCBs and aspirin were the most frequently prescribed medications. The mean 1-year direct medical cost was estimated to be USD$19969 per patient, with in-patient hospitalization being the major cost driver. Comorbid state with hypertension, diabetes or renal impairment increased the cost of care and the increments reached statistical significance for diabetes and renal impairment. Humanistic outcome showed promising improvement in quality of life with the local HFpEF management approach. In view of the improvement in the prognosis of HFrEF due to advances in evidence-based therapies yet little progress in those of HFpEF, the latter may become the predominant form of clinical HF in the near future, especially with the aging population in Hong Kong. The present study provided information on the local practice and the substantial economic burden incurred by HFpEF. It conveyed the need to unify every effort to improve HFpEF management, and to reduce the associated healthcare expenditure by rational resource allocation and development of cost-saving strategies.
